# BUILDING FORTITUDE: STRATEGIES FOR TEACHING TENDER TOPICS IN GERONTOLOGY CURRICULA

**DOI:** 10.1093/geroni/igae098.0880

**Published:** 2024-12-31

**Authors:** Autumn Decker, Raven Weaver, Laura Donorfio

**Affiliations:** Chair; Co-Chair; Discussant

## Abstract

Gerontology educators are tasked with navigating a myriad of sensitive topics (e.g., discrimination/inequality, abuse/neglect, end-of-life) that lay the foundation for understanding aging and its complexities over the life course. These topics challenge educators but hold profound significance for gerontological practice. Inequities shape opportunities over time as advantages/disadvantages accumulate, such that “the reality is later life is not the end of inequality, it is inequality’s end game” (Abramson, 2016, p. 4). Inequities related to racism, ageism, and homo/transphobia affect individuals’ exposure to death, trauma, loss, and grief, and require facilitation of difficult classroom conversations. Social justice necessitates learning how to provide all groups with adequate supports/resources for growing old and educators are responsible for imbuing the next generation of gerontological practitioners with knowledge, skills, and compassion. Presenters in this symposium delve into the nuances of navigating “tender topics” in gerontology curricula. Two presentations share research on end-of-life teaching strategies from a faculty perspective (Decker and colleagues) and from an interdisciplinary team approach (Newsham and colleagues). Weaver and colleagues present insights on suicide education based on faculty/student perspectives. Finally, Bolkan and colleagues provide survey data on student perceptions of sensitive issues and an overview of effective pedagogies for teaching difficult topics. Each presenter offers unique insights, findings, and tools to foster inclusive learning environments and lead challenging discussions with integrity and fortitude. Following the presentations, Laura Donorfio, AGHE Vice-Chair Elect and creator of the AGHE Teaching Institute will serve as discussant to identify key themes and facilitate a discussion with attendees.

